# Smoking and Drinking Habits Five Years after Baseline in the JACC Study

**DOI:** 10.2188/jea.15.S56

**Published:** 2005-05-16

**Authors:** Miyuki Kawado, Sadao Suzuki, Shuji Hashimoto, Shinkan Tokudome, Takesumi Yoshimura, Akiko Tamakoshi

**Affiliations:** 1 Department of Hygiene, Fujita Health University School of Medicine.; 2 Department of Health Promotion and Preventive Medicine, Nagoya City University Graduate School of Medical Sciences.; 3 Fukuoka Institute of Health and Environmental Sciences.; 4 Department of Preventive Medicine/Biostatistics and Medical Decision Making, Nagoya University Graduate School of Medicine.

**Keywords:** smoking, alcohol consumption, cohort study

## Abstract

BACKGROUND: Observing longitudinal changes in smoking and drinking habits is important for evaluating the risk of incidence and death from cancer or other diseases in a cohort study.

METHODS: Smoking and drinking habits at baseline and about five years later among 18,312 males and 28,338 females were obtained from the baseline and interim surveys in the Japan Collaborative Cohort Study for Evaluation of Cancer Risk (JACC Study) sponsored by Monbusho (Ministry of Education, Science, Sports and Culture of Japan). Changes in smoking and drinking habits between the two surveys were observed. Odds ratios for quitting these habits at interim survey were estimated.

RESULTS: Percentages of current smokers at baseline and interim surveys were 51.0% and 45.5% in males, and 5.2% and 4.8% in females, respectively. Percentages of current drinkers at baseline and interim surveys were 78.0% and 73.2% in males, and 29.5% and 23.5% in females, respectively. The number of cigarettes per day among male current smokers and the usual amount of alcohol consumed on each occasion among current drinkers decreased between the two surveys. Odds ratios for smoking cessation increased with age at baseline and decreased with the number of cigarettes per day at baseline. Odds ratios for drinking cessation increased with age at baseline and decreased with the usual amount of alcohol consumed on each occasion at baseline.

CONCLUSION: The decrease in smoking and drinking habits was observed during the five-year follow-up period. Higher age and lower levels of exposure were associated with quitting smoking or drinking.

Changes in the habits of smokers and heavy drinkers, especially the problem of quitting, are important for the prevention of cancer and other diseases.^1^^-^^4^ Cross-sectional studies of smoking or drinking habits have been reported in many countries including Japan.^5^^-^^12^ However, longitudinal changes in smoking and drinking habits and their related factors have also been investigated,^13^^-^^19^ but such few studies have been conducted among Japanese.^20^

If exposure data in cohort studies are only measured at baseline even though exposure histories change significantly after baseline, the estimates of relative risk may be biased due to insufficient information regarding exposure.^21^ To evaluate the relative risk of smoking and drinking habits in such cohort studies, it is important to clarify the extent of such changes during the follow-up.

The Japan Collaborative Cohort Study for Evaluation of Cancer Risk (JACC Study) sponsored by Monbusho (Ministry of Education, Science, Sports and Culture of Japan)^22^ is a large-scale cohort study surveying the associations between cancer risk and lifestyle and living conditions. An interim survey covering half of the cohort subjects was conducted about five years after the baseline survey.

Among subjects in the JACC Study, we observed the changes in smoking and drinking habits from the baseline survey to the interim survey, and analyzed characteristics of smokers and drinkers at the baseline survey associated with smoking and drinking cessation during the two surveys.

## METHODS

### Subjects

The baseline survey of the JACC Study was conducted in 45 areas from 1988 to 1990, and 46,465 males and 64,327 females aged 40 to 79 years completed a baseline questionnaire. The interim survey was conducted in 31 areas (it was asked to every participant in 18 areas and asked to some of the participants in 13 areas) from 1993 to 1995 using the questionnaire of the baseline survey with some modifications, while not conducted in 14 areas. Total 46,650 participants (18,312 males and 28,338 females) completed the questionnaire. For 18 areas in which the interim survey was conducted in every participant of baseline survey, 37,853 of 48,016 baseline participants were responded, and the response rate was 78.8%. For 13 areas in which the survey was conducted in part of the participants, 8,797 of 36,460 participants were responded, and the response rate was 24.1%. The mean interval between the baseline and interim surveys was 5.0 (standard deviation = 0.9) years.

Subjects for our analysis of smoking habits reported here were restricted to 16,778 males and 21,161 females who answered a question regarding smoking status in both the baseline and interim survey questionnaires. Similarly, subjects for the analysis of drinking habits were restricted to 16,567 males and 22,303 females who responded to a question about their drinking status. Table 1 shows the numbers of these subjects by sex and age at the baseline survey.

**Table 1.** Number of subjects by sex and age at baseline survey.

Sex	Age (years) at baseline survey		Number of subjects [%]			

			for smoking habit*		for drinking habit^†^	
Male	40-44		2,045	[12.2]	2,010	[12.1]
	45-49		2,020	[12.0]	2,004	[12.1]
	50-54		2,296	[13.7]	2,281	[13.8]
	55-59		3,076	[18.3]	3,019	[18.2]
	60-64		3,433	[20.5]	3,408	[20.6]
	65-69		1,979	[11.8]	1,936	[11.7]
	70-74		1,308	[7.8]	1,272	[7.7]
	75-79		621	[3.7]	637	[3.8]

		Total	16,778	[100.0]	16,567	[100.0]

Female	40-44		2,679	[12.7]	2,695	[12.1]
	45-49		2,931	[13.9]	2,980	[13.4]
	50-54		3,251	[15.4]	3,326	[14.9]
	55-59		3,768	[17.8]	3,925	[17.6]
	60-64		3,941	[18.6]	4,225	[18.9]
	65-69		2,591	[12.2]	2,855	[12.8]
	70-74		1,296	[6.1]	1,514	[6.8]
	75-79		704	[3.3]	783	[3.5]

		Total	21,161	[100.0]	22,303	[100.0]

[ ]: column %

* Subjects completing a question about smoking status in both baseline and interim surveys

† Subjects completing a question about drinking status in both baseline and interim surveys

### Smoking and drinking habits

The self-administered questionnaires of the baseline and interim surveys included the same questions about smoking and drinking habits. The questions about smoking habits were with regard to smoking status (“current smoker,” “former smoker” and “never smoked”), age when starting smoking, and the number of cigarettes per day. The questions regarding drinking habit concerned drinking status (“current drinker,” “former drinker” and “never drank”), age when starting drinking, frequency of drinking per week (“less than 1 day,” “1-2 days,” “3-4 days” and “everyday”), and the usual amount of alcohol consumed on each occasion. The unit of alcohol consumption per occasion was “gou” (Japanese standard unit for an alcoholic beverage), which can be converted to 23 g units of ethanol.

### Statistical analysis

Changes were examined in smoking status, the number of cigarettes per day, drinking status, drinking frequency, and alcohol consumption per occasion between the baseline and interim surveys. Smoking status at the interim survey was re-categorized into “current smoker” (“current smoker”) and “nonsmoker” (“never smoked” or “former smoker”). Similarly, alcohol drinking status at interim survey was re-categorized into “current drinker” and “nondrinker” (“never drunk” or “former drinker”). The number of cigarettes per day was categorized as 1-9, 10-19, 20-29, 30-39 or 40 and over. Alcohol consumption per each occasion (gou) was categorized as 0.1-0.9, 1.0-1.9, 2.0-2.9 or 3.0 and over.

Smoking cessation percentages at the interim survey among current smokers from the baseline survey were observed by using each baseline characteristic; i.e., age at the baseline survey, age when starting smoking and the number of cigarettes per day. Logistic regression analysis was used to estimate the odds ratios for smoking cessation at interim survey among current smokers at baseline using these baseline characteristics as independent variables (as dummy variables).

Drinking cessation percentages at the interim surveys among current drinkers from the baseline survey were observed by using each baseline characteristic; i.e., age at the baseline survey, age when starting drinking, drinking frequency and alcohol consumption per occasion. Logistic regression analysis was used to estimate the odds ratios for drinking cessation at interim survey among current smokers at baseline using these baseline characteristics as independent variables (as dummy variables).

All analyses were conducted using SAS^®^ software, version 8.2 (SAS Institute, Inc., Cary, NC, USA).^22^

### Ethical review

Our entire study design, which comprised singular and collective use of epidemiologic data and biological materials (serum only), was approved in 2000 by the Ethical Board at Nagoya University School of Medicine, where the central secretariat of the JACC study is located.

## RESULTS

### Changes in smoking habits

**Figure 1.**
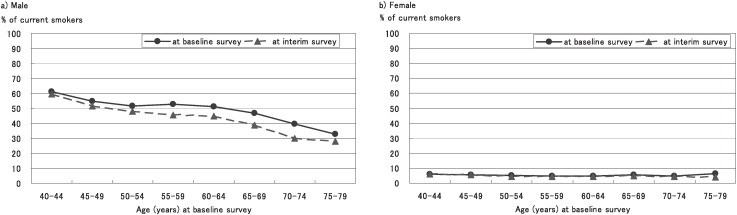
Sex- and age-specific percentages of current smokers at baseline and interim surveys.

Figure 1 shows the percentages of current smokers at the baseline and interim surveys by sex and age (at the baseline survey) group. In males, those percentages were 51.0% at the baseline survey and 45.5% at the interim survey. In females, the percentages of current smokers were 5.2% in the baseline survey against 4.8% at the interim survey. The percentages in the interim survey were lower than those in the baseline survey in every sex and age group except for the female 40 to 44-years-old group (increased 5.9% to 6.2%).

Table 2 shows changes in smoking status between the two surveys. In males, the percentages of current smokers at the interim survey among current smokers, former smokers and never smokers at the baseline survey were 83.9%, 7.6% and 2.8%, respectively, against 78.3%, 12.8% and 0.5%, respectively, in females.

**Table 2.** Changes in smoking status between baseline and interim surveys.

Sex	Smoking status at baseline survery		Smoking status at interim survey				Total	

			Current smoker		Nonsmoker			
Male	Current smoker		7,181	(83.9)	1,382	(16.1)	8,563	[51.0]
	Former smoker		362	(7.6)	4,383	(92.4)	4,745	[28.3]
	Never smoked		98	(2.8)	3,372	(97.2)	3,470	[20.7]

		Total	7,641	(45.5)	9,137	(54.5)	16,778	(100.0)

Female	Current smoker		864	(78.3)	240	(21.7)	1,104	[5.2]
	Former smoker		47	(12.8)	321	(87.2)	368	[1.7]
	Never smoked		97	(0.5)	19,592	(99.5)	19,689	[93.0]

		Total	1,008	(4.8)	20,153	(95.2)	21,161	(100.0)

( ): row %; [ ]: column %

Table 3 shows changes in the number of cigarettes per day among current smokers at the baseline and interim surveys who responded to the question. In males, the percentage of those smoking 1-19 cigarettes per day at the interim survey was higher than at the baseline survey, whereas that of those smoking 30 or more per day at the interim survey was lower than at the baseline survey. In females, however, the percentages of those smoking 1-19 cigarettes per day at the interim survey was slightly lower than at the baseline survey, whereas that of those smoking 30 or more per day at the interim survey was slightly higher than at the baseline survey.

**Table 3.** Changes in number of cigarettes per day between baseline and interim surveys among current smokers at both surveys.

Sex	Number of cigatettes per day at baseline survey		Number of cigarettes per day at interim survey										Total	

			1-9		10-19		20-29		30-39		40 and over			
Male	1-9		141	(56.2)	93	(37.1)	13	(56.2)	2	(0.8)	2	(0.8)	251	(3.6)
	10-19		148	(8.0)	1,290	(69.5)	393	(8.0)	17	(0.9)	9	(0.5)	1,857	(26.8)
	20-29		57	(1.7)	571	(17.3)	2,319	(1.7)	289	(8.8)	61	(1.9)	3,297	(47.6)
	30-39		7	(0.7)	37	(3.9)	389	(0.7)	394	(41.7)	118	(12.5)	945	(13.7)
	40 and over		4	(0.7)	7	(1.2)	91	(0.7)	146	(25.5)	324	(56.6)	572	(8.3)

		Total	357	(5.2)	1,998	(28.9)	3,205	(5.2)	848	(12.3)	514	(7.4)	6,922	(100.0)

Female	1-9		123	(77.8)	31	(19.6)	3	(1.9)	0	(0.0)	1	(0.6)	158	(19.1)
	10-19		45	(11.1)	290	(71.8)	67	(16.6)	1	(0.2)	1	(0.2)	404	(48.7)
	20-29		2	(0.9)	46	(21.1)	151	(69.3)	17	(7.8)	2	(0.9)	218	(26.3)
	30-39		0	(0.0)	0	(0.0)	6	(25.0)	13	(54.2)	5	(20.8)	24	(2.9)
	40 and over		1	(4.0)	0	(0.0)	5	(20.0)	5	(20.0)	14	(56.0)	25	(3.0)

		Total	171	(20.6)	367	(44.3)	232	(28.0)	36	(4.3)	23	(2.8)	829	(100.0)

( ): row %; [ ]: column %

### Changes in drinking habits

**Figure 2.**
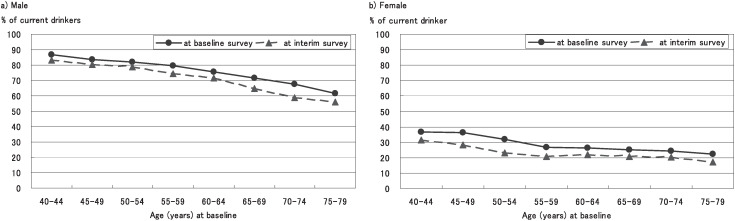
Sex- and age-specific percentages of current drinkers at baseline and interim surveys.

Figure 2 shows the percentages of current drinkers at the baseline and interim surveys by sex and age (at the baseline survey) group. In males, the percentage of current drinkers was 78.0% at the baseline survey and 73.2% at the interim survey. In females, the corresponding percentages were 29.5% and 23.5%, respectively. The percentage of current drinkers at the interim survey was lower than at the baseline survey in every age group.

Table 4 shows changes in drinking status between the baseline and interim surveys. In males, the percentages of drinkers at the interim survey among current drinkers, former drinkers and never drinkers at the baseline survey were 88.7%, 38.6% and 11.8%, respectively, against 59.4%, 35.3% and 7.8%, respectively, in females.

**Table 4.** Changes in drinking status between baseline and interim surveys.

Sex	Drinking status at baseline survey		Drinking status at interim survey				Total	

			Current drinker		Nondrinker			
Male	Current drinker		11,457	(88.7)	1,457	(11.3)	12,914	[78.0]
	Former drinker		350	(38.6)	556	(61.4)	906	[5.5]
	Never drank		323	(11.8)	2,424	(88.2)	2,747	[16.6]

		Total	12,130	(73.2)	4,437	(26.8)	16,567	(100.0)

Female	Current drinker		3,905	(59.4)	2,665	(40.6)	6,570	[29.5]
	Former drinker		134	(35.3)	246	(64.7)	380	[1.7]
	Never drank		1,200	(7.8)	14,153	(92.2)	15,353	[68.8]

		Total	5,239	(23.5)	17,064	(76.5)	22,303	(100.0)

( ): row %; [ ]: column %

Table 5 shows changes in drinking frequency between the baseline and interim surveys among current drinkers at both surveys who responded to this question. The distribution of drinking frequency at the interim survey was nearly equal to that at the baseline survey.

**Table 5.** Changes in drinking frequency per week between baseline and interim surveys among current drinkers at both surveys.

Sex	Frequency of drinking per week at baseline survey		Frequency of drinking per week at interim survey								Total	

			Less than 1 day		1-2 days		3-4 days		Everyday			
Male	Less than 1 day		116	(38.7)	86	(28.7)	31	(10.3)	67	(22.3)	300	[3.3]
	1-2 days		98	(15.7)	225	(36.1)	168	(26.9)	133	(21.3)	624	[6.9]
	3-4 days		49	(4.3)	151	(13.2)	427	(37.2)	520	(45.3)	1,147	[12.8]
	Everyday		60	(0.9)	138	(2.0)	535	(7.7)	6,188	(89.4)	6,921	[77.0]

		Total	323	(3.6)	600	(6.7)	1,161	(12.9)	6,908	(76.8)	8,992	(100.0)

Female	Less than 1 day		212	(42.0)	165	(32.7)	88	(17.4)	40	(7.9)	505	[16.4]
	1-2 days		193	(24.3)	297	(37.4)	198	(24.9)	106	(13.4)	794	[25.8]
	3-4 days		64	(8.7)	199	(27.0)	272	(37.0)	201	(27.3)	736	[23.9]
	Everyday		33	(3.2)	84	(8.1)	210	(20.2)	713	(68.6)	1,040	[33.8]

		Total	502	(16.3)	745	(24.2)	768	(25.0)	1,060	(34.5)	3,075	(100.0)

( ): row %; [ ]: column %

Table 6 shows changes in the usual amount of alcohol consumed on each occasion between the baseline and interim surveys among current drinkers at both surveys who responded to this question. The percentage of those consuming 0.1-0.9 gou per occasion at the interim survey was higher than at the baseline survey in both males and females.

**Table 6.** Changes in usual amount of alcohol consumed on each occasion between baseline and interim surveys among current drinkers at both surveys.

Sex	Usual amount of alcohol consumed on each occasion at baseline survey (gou*)		Usual amount of alcohol consumed on each occasion at interim survey (gou*)								Total	

			0.1-0.9		1.0-1.9		2.0-2.9		3.0 and over			
Male	0.1-0.9		414	(51.5)	247	(30.7)	95	(11.8)	48	(6.0)	804	[9.6]
	1.0-1.9		663	(19.4)	1,857	(54.4)	758	(22.2)	134	(3.9)	3,412	[40.9]
	2.0-2.9		109	(3.8)	655	(22.8)	1,650	(57.4)	463	(16.1)	2,877	[34.5]
	3.0 and over		35	(2.8)	119	(9.5)	473	(37.8)	625	(49.9)	1,252	[15.0]

		Total	1,221	(14.6)	2,878	(34.5)	2,976	(35.7)	1,270	(15.2)	8,345	(100.0)

Female	0.1-0.9		1,040	(85.9)	149	(12.3)	15	(1.2)	7	(0.6)	1,211	[53.8]
	1.0-1.9		405	(47.6)	370	(43.5)	64	(7.5)	12	(1.4)	851	[37.8]
	2.0-2.9		30	(22.2)	50	(37.0)	44	(32.6)	11	(8.1)	135	[6.0]
	3.0 and over		14	(25.0)	12	(21.4)	10	(17.9)	20	(35.7)	56	[2.5]

		Total	1,489	(66.1)	581	(25.8)	133	(5.9)	50	(2.2)	2,253	(100.0)

( ): row %; [ ]: column %

* 1 gou = 23 g of ethanol

### Characteristics of smokers associated with smoking cessation

Table 7 shows the percentages of smoking cessation at the interim survey among current smokers at the baseline survey by age at the baseline survey, age when starting smoking, and the number of cigarettes per day. The percentages of those who quit increased with age at the baseline survey and age when starting smoking, but decreased with the number of cigarettes per day.

**Table 7.** Smoking cessation percentages at interim survey among current smokers at baseline survey by characteristics of current smokers at baseline survey.

Variable		Male			Female		
	
		Number of subjects	Smoking cessation at interim survey		Number of subjects	Smoking cessation at interim survey	
Age at baseline survey	40-44	1,253	121	(9.7)	158	21	(13.3)
	45-49	1,103	115	(10.4)	167	30	(18.0)
	50-54	1,181	145	(12.3)	163	47	(28.8)
	55-59	1,619	278	(17.2)	182	36	(19.8)
	60-64	1,762	317	(18.0)	181	34	(18.8)
	65-69	925	202	(21.8)	143	34	(23.8)
	70-74	516	153	(29.7)	64	14	(21.9)
	75-79	204	51	(25.0)	46	24	(52.2)

Age when starting smoking	0-19	1,610	217	(13.5)	55	8	(14.5)
	20-24	4,914	746	(15.2)	221	31	(14.0)
	25-29	910	181	(19.9)	162	23	(14.2)
	30-34	352	68	(19.3)	185	33	(17.8)
	35-39	73	18	(24.7)	111	22	(19.8)
	40 and over	138	37	(26.8)	276	75	(27.2)
	Unknown	566	115	(20.3)	94	48	(51.1)

Number of cigarettes per day	1-9	394	126	(32.0)	252	89	(35.3)
	10-19	2,379	470	(19.8)	504	89	(17.7)
	20-29	3,930	550	(14.0)	253	30	(11.9)
	30-39	1,106	142	(12.8)	30	4	(13.3)
	40 and over	650	68	(10.5)	27	2	(7.4)
	Unknown	104	26	(25.0)	38	26	(68.4)

( ): row %

Table 8 shows the odds ratios for smoking cessation at the interim survey by the characteristics of current smokers at the baseline survey. In males, the odds ratio compared with 40 to 44-years-old at baseline survey was 1.0-3.3, which increased with age at the baseline survey. The odds ratio compared with 20 to 24-years-old at starting smoking was 1.0-1.7 in the other groups of age when starting smoking. The odds ratio decreased with the number of cigarettes per day. In females, the corresponding odds ratio compared with those 40 to 44-years-old was 4.8 in the 75 to 79-years-old group at baseline, and 1.1-2.2 in the other age groups. The odds ratio compared with 20 to 24-years-old when starting smoking was 0.9-1.7 in the other groups of age when starting smoking. The odds ratio compared with those smoking 1-9 cigarettes per day was less than 0.4 in those smoking 20-29 and those smoking 40 cigarettes and over per day.

**Table 8.** Odds ratios for smoking cessation at interim survey among current smokers at baseline survery by characteristics of current smokers at baseline survey.

Variable		Male		Female	
	
		Odds ratio	p value	Odds ratio	p value
Age at baseline survey	40-44	1.00		1.00	
	45-49	1.04	0.754	1.11	0.762
	50-54	1.23	0.158	2.15	0.023
	55-59	1.82	<0.001	1.34	0.380
	60-64	1.81	<0.001	1.12	0.744
	65-69	2.31	<0.001	1.67	0.129
	70-74	3.33	<0.001	1.27	0.581
	75-79	2.21	<0.001	4.83	<0.001

Age when starting smoking	0-19	1.02	0.814	0.99	0.973
	20-24	1.00		1.00	
	25-29	1.29	0.024	0.94	0.887
	30-34	1.14	0.414	1.21	0.675
	35-39	1.64	0.086	1.31	0.570
	40 and over	1.73	0.010	1.65	0.243

Number of cigarettes per day	1- 9	1.00		1.00	
	10-19	0.57	<0.001	0.47	<0.001
	20-29	0.43	<0.001	0.31	<0.001
	30-39	0.44	<0.001	0.49	0.209
	40 and over	0.37	<0.001	0.22	0.044

### Characteristics of drinkers associated with drinking cessation

Table 9 shows the percentages of drinking cessation at the interim survey among current drinkers at the baseline survey by age at the baseline survey, age when starting drinking, drinking frequency at the baseline survey, and the usual amount of alcohol consumed on each occasion at the baseline survey. The drinking cessation percentages increased with age at the baseline survey in males, but showed no major change with age at the baseline survey in females. It also showed no major change with age when starting drinking, and decreased with drinking frequency and the usual amount of alcohol consumed on each occasion at the baseline survey.

**Table 9.** Drinking cessation percentages at interim survey among current drinkers at baseline survey by characteristics of current drinkers at baseline suvey.

Variable		Male			Female		
	
		Number of subjects	Drinking cessation at interim survey		Number of subjects	Drinking cessation at interim survey	
Age at baseline survey	40-44	1,741	123	(7.1)	991	342	(34.5)
	45-49	1,674	141	(8.4)	1,086	424	(39.0)
	50-54	1,869	157	(8.4)	1,064	486	(45.7)
	55-59	2,404	276	(11.5)	1,050	465	(44.3)
	60-64	2,583	310	(12.0)	1,117	441	(39.5)
	65-69	1,389	199	(14.3)	717	280	(39.1)
	70-74	861	169	(19.6)	370	147	(39.7)
	75-79	393	82	(20.9)	175	80	(45.7)


Age when starting drinking	0-19	490	34	(6.9)	49	14	(28.6)
	20-24	2,553	215	(8.4)	360	111	(30.8)
	25-29	1,438	87	(6.1)	178	49	(27.5)
	30-34	1,913	108	(5.6)	469	118	(25.2)
	35-39	575	41	(7.1)	266	62	(23.3)
	40 and over	1,977	152	(7.7)	1,823	522	(28.6)
	Unknown	3,968	820	(20.7)	3,425	1,789	(52.2)


Frequency of drinking per week	Less than 1 day	806	435	(54.0)	2,001	1,313	(65.6)
	1-2 days	1,100	310	(28.2)	1,693	694	(41.0)
	3-4 days	1,639	178	(10.9)	1,129	269	(23.8)
	Everyday	8,832	448	(5.1)	1,396	224	(16.0)
	Unknown	537	86	(16.0)	351	165	(47.0)


Usual amount of alcohol consumed on each occasion (gou*)	0.1-0.9	1,039	180	(17.3)	1,974	667	(33.8)
	1.0-1.9	3,882	357	(9.2)	1,210	280	(23.1)
	2.0-2.9	3,093	117	(3.8)	171	28	(16.4)
	3.0 and over	1,345	49	(3.6)	71	9	(12.7)
	Unknown	3,555	754	(21.2)	3,144	1,681	(53.5)

( ): row %

* 1 gou = 23g of ethanal

Table 10 shows the odds ratios for drinking cessation between the baseline and interim surveys by the characteristics of current drinkers at the baseline survey. In males, the odds ratio compared with 40 to 44-years-old at baseline survey was 0.9 in the 45 to 49-years-old age group and 1.6-3.3 in the other age group, which increased with age at the baseline survey. That ratio compared with 20 to 24-years-old when starting drinking was 0.8-1.0 in the other groups of age when starting drinking. The odds ratio decreased with drinking frequency and usual amount of alcohol consumed on each occasion. In females, the odds ratio compared with 40 to 44-year olds was 1.0-2.5 in the other age groups at the baseline survey. The odds ratio compared with those 20 to 24-years-old when starting drinking was 0.9-1.2 in the other age groups when starting drinking, and it decreased with drinking frequency and the usual amount of alcohol consumed on each occasion.

**Table 10.** Odds ratios for drinking cessation at interim survey among current drinkers at baseline survery by characteristics of current drinkers at baseline survey.

Variable		Male		Female	
	
		Odds ratio	p value	Odds ratio	p value
Age at baseline survey	40-44	1.00		1.00	
	45-49	0.87	0.550	1.54	0.020
	50-54	1.59	0.035	2.28	<0.001
	55-59	2.02	<0.001	1.81	0.002
	60-64	2.26	<0.001	1.73	0.004
	65-69	2.33	<0.001	1.80	0.006
	70-74	3.03	<0.001	1.00	0.994
	75-79	3.27	<0.001	2.46	0.008

Age when starting drinking	0-19	0.90	0.626	0.98	0.953
	20-24	1.00		1.00	
	25-29	0.88	0.380	1.17	0.535
	30-34	0.78	0.068	0.86	0.437
	35-39	0.98	0.907	1.05	0.834
	40 and over	0.76	0.036	1.17	0.360

Freqency of drinking per week	Less than 1 day	7.81	<0.001	7.44	<0.001
	1-2 days	5.05	<0.001	3.72	<0.001
	3-4 days	1.87	<0.001	2.07	<0.001
	Everyday	1.00		1.00	

Usual amount of alcohol consumed on each occasion (gou*)	0.1-0.9	1.00		1.00	
	1.0-1.9	0.65	<0.001	0.63	<0.001
	2.0-2.9	0.38	<0.001	0.40	0.001
	3.0 and over	0.39	<0.001	0.29	0.021

* 1 gou = 23g of ethanol

## DISCUSSION

The percentages of current smokers decreased more at the interim than at the baseline survey. As previous cross-sectional and longitudinal studies have reported, the decrease is associated with aging of the population.^13^^,^^14^ A similar decrease in smoking habits due to aging might occur in the whole population of the JACC study during the follow-up period. Higher age and fewer cigarettes per day at the baseline survey were associated with smoking cessation during about five years of the follow-up period. A positive correlation between increasing age and smoking cessation has been discussed in other studies.^9^^,^^10^ The higher incidence of smoking-related diseases in older age might persuade some smokers to adapt a healthy lifestyle, thus making it easier for them to quit. A trend toward an increased number of cigarettes per day is one of the criteria for nicotine dependence.^24^ Because of such a dependence, quitting smoking may be more difficult for heavy users who smoke more cigarettes per day.^24^ This relationship is also consistent with that in other studies.^13^^,^^15^

The percentage of current drinkers was lower at the interim survey than at baseline. Several longitudinal studies have shown a similar decline in alcohol intake with aging,^16^^,^^17^ a decline which may well have occurred in the whole population of the JACC Study. Age at baseline, frequency of drinking, and usual amount of alcohol consumed on each occasion were associated with drinking cessation. In many cross-sectional and some longitudinal studies, lower levels of alcohol consumption (including quitting drinking entirely), were observed among older subjects.^16^ Deteriorating health may also be related to this trend among the elderly.^18^ A negative correlation between the level of consumption and drinking cessation during the follow-up period has been reported in many studies.^16^

It has been well-known that nondifferential misclassification caused underestimates of relative risk. The changes in smoking and drinking habits during the follow-up period in a cohort study might affect their estimates of the relative risk of incidence of disease and death from cancer or other diseases if only the exposure data at baseline were used.^19^^,^^20^ However, the risk of cancer or other chronic diseases would be affected by long-term exposure to smoking or drinking, and this effect might not be instantly affected by the changes in these habits during the follow-up period. In addition, differences in the percentages of current smokers or drinkers in the whole population in the baseline and interim surveys were less than 5%. Therefore, estimates of the relative risk of smoking and drinking based only on baseline exposure data in this population would not have much of an effect on the study conclusions, despite a possible bias toward underestimation due to the lack of exposure information after baseline.

## MEMBER LIST OF THE JACC STUDY GROUP

The present investigators involved, with the co-authorship of this paper, in the JACC Study and their affiliations are as follows: Dr. Akiko Tamakoshi (present chairman of the study group), Nagoya University Graduate School of Medicine; Dr. Mitsuru Mori, Sapporo Medical University School of Medicine; Dr. Yutaka Motohashi, Akita University School of Medicine; Dr. Ichiro Tsuji, Tohoku University Graduate School of Medicine; Dr. Yosikazu Nakamura, Jichi Medical School; Dr. Hiroyasu Iso, Institute of Community Medicine, University of Tsukuba; Dr. Haruo Mikami, Chiba Cancer Center; Dr. Yutaka Inaba, Juntendo University School of Medicine; Dr. Yoshiharu Hoshiyama, University of Human Arts and Sciences; Dr. Hiroshi Suzuki, Niigata University School of Medicine; Dr. Hiroyuki Shimizu, Gifu University School of Medicine; Dr. Hideaki Toyoshima, Nagoya University Graduate School of Medicine; Dr. Kenji Wakai, Aichi Cancer Center Research Institute; Dr. Shinkan Tokudome, Nagoya City University Graduate School of Medical Sciences; Dr. Yoshinori Ito, Fujita Health University School of Health Sciences; Dr. Shuji Hashimoto, Fujita Health University School of Medicine; Dr. Shogo Kikuchi, Aichi Medical University School of Medicine; Dr. Akio Koizumi, Graduate School of Medicine and Faculty of Medicine, Kyoto University; Dr. Takashi Kawamura, Kyoto University Center for Student Health; Dr. Yoshiyuki Watanabe, Kyoto Prefectural University of Medicine Graduate School of Medical Science; Dr. Tsuneharu Miki, Graduate School of Medical Science, Kyoto Prefectural University of Medicine; Dr. Chigusa Date, Faculty of Human Environmental Sciences, Mukogawa Women’s University ; Dr. Kiyomi Sakata, Wakayama Medical University; Dr. Takayuki Nose, Tottori University Faculty of Medicine; Dr. Norihiko Hayakawa, Research Institute for Radiation Biology and Medicine, Hiroshima University; Dr. Takesumi Yoshimura, Fukuoka Institute of Health and Environmental Sciences; Dr. Akira Shibata, Kurume University School of Medicine; Dr. Naoyuki Okamoto, Kanagawa Cancer Center; Dr. Hideo Shio, Moriyama Municipal Hospital; Dr. Yoshiyuki Ohno, Asahi Rosai Hospital; Dr. Tomoyuki Kitagawa, Cancer Institute of the Japanese Foundation for Cancer Research; Dr. Toshio Kuroki, Gifu University; and Dr. Kazuo Tajima, Aichi Cancer Center Research Institute.
